# A descriptive review of the methodologies used in household surveys on medicine utilization

**DOI:** 10.1186/1472-6963-8-222

**Published:** 2008-10-31

**Authors:** Andréa D Bertoldi, Aluísio JD Barros, Anita Wagner, Dennis Ross-Degnan, Pedro C Hallal

**Affiliations:** 1 Programa de pós-graduação em Saúde Coletiva, Universidade do Vale do Rio dos Sinos, Brazil; 2 Takemi Program in International Health, Harvard School of Public Health, Boston, USA; 3 Programa de Pós-graduação em Epidemiologia, Universidade Federal de Pelotas, Brasil; 4 Department of Ambulatory Care and Prevention, Harvard Medical School and Harvard Pilgrim Health Care, Boston, USA

## Abstract

**Background:**

Studies carried out in the community enable researchers to understand access to medicines, affordability, and barriers to use from the consumer's point of view, and may stimulate the development of adequate medicines policies. The aim of the present article was to describe methodological and analytical aspects of quantitative studies on medicine utilization carried out at the household level.

**Methods:**

Systematic review of original papers with data collected in studies in which the household was a sampling unit, published between 1995 and 2008. The electronic review was carried out in Medline/Pubmed, Scielo and Lilacs. The reference lists of the papers identified were examined, as well as other publications by their authors. Studies on the utilization of specific pharmacological groups, or those including only respondents with a given disease were excluded.

**Results:**

Out of 4852 papers initially identified in the literature search, 61 fulfilled our inclusion criteria. Most studies were carried out in Europe and North America and used a cross-sectional approach. More than 80% used face-to-face interviews for data collection, and the most frequently used recall period for assessing medicine utilization was 14–15 days. In 59% of the studies, interviewers were trained to request the packaging of the medicines reported by the subjects; medical prescriptions were requested less frequently (15% of the studies).

**Conclusion:**

These data will be useful for updating researchers on what methods their peers are currently using. Such information may help overcome challenges in the planning and analyses of future studies. Moreover, this publication may contribute to the improvement of the quality of medicine use data obtained in household surveys.

## Background

Studies on the overall prevalence of medicine utilization are frequent in the scientific literature. Such studies are characterized by the investigation of any medicine used within a defined period of time. These studies represent an important tool to be used in health system evaluation.[1] Information may help draw conclusions about prevalence of diseases, treatment of adverse events, health status and quality of health care or of prescribers' behavior.[2] Studies carried out in the community also enable researchers to understand medicine use and its related aspects from the consumer's point of view, and may stimulate the development of adequate medicines policies.[3] This type of study may also help in understanding access to medicines, affordability, and barriers to use, at the community level.

In low and middle-income countries, in general, there are no computerized systems that integrate the information about medicines. Therefore, in most cases, it is not possible to carry out medicine utilization studies based on this source of information. As a result, researchers usually rely on studies with direct interviews with consumers, or investigate medical records, which are often incomplete. Taking into account that studies based on questionnaires are subject to reporting bias, it is important to use a carefully designed methodology for data collection.

The primary aim of this review is to describe the methodologies used for collecting and analyzing quantitative data on medicines use in household surveys. The paper also analyzes qualitatively the methodologies employed in the reviewed studies, and provides recommendations for future studies in the field.

## Methods

A literature review was carried out using the Pubmed, Scielo and Lilacs databases. The following keywords were searched in the title or abstract of the papers: "drug(s) use", "drug(s) utilization", "drug(s) utilisation", "drug(s) usage", "drug(s) consumption", "medication use", "medication utilization", "medication usage", "medication consumption", "medicine(s) use", "medicine usage", "medicine consumption", "self-medication", "over-the-counter".

Several restriction criteria were used in the electronic search: (a) only articles published in Portuguese, English and Spanish were considered (only four papers were excluded due to this criterion); (b) only studies published between January 1995 and June 2008 were included, in order to focus on a description of the survey methods in current use; (c) only original papers were included. Studies in hospitals, schools, universities, nurseries, pharmacy counters, health clinics, and workplaces were excluded, as well as those including only institutionalized individuals (nursing homes and prisons). Finally, studies investigating the specific use of a given pharmacological group or including only individuals with a given morbidity were not considered.

Using the keywords mentioned earlier in Pubmed, 4852 titles were found until July 2005. After reviewing their titles and abstracts, 134 original papers appeared to fulfill the inclusion criteria, and therefore, their full texts were obtained. From July 2005 to June 2008, the same search strategy was automatically repeated in Pubmed. The searches at Scielo and Lilacs added few publications, and were made in July 2005, April 2006 and June 2008. Articles detected in the electronic search were reviewed by two of the authors, who decided whether or not the manuscript fulfilled the inclusion criteria. When they disagreed, the article was discussed by the two until a final decision was reached, based on the inclusion and exclusion criteria.

The references of the selected papers were reviewed in order to find other eligible publications. A search by the names of all first authors from the selected papers was also made. In seven cases, in which more than one publication was based on the same data, only the first was included in our review. After a detailed examination of the selected papers (obtained in full), 61 fulfilled all inclusion criteria.

The original papers included in the review were classified according to several characteristics:

- Country of data collection;

- Continent of data collection: studies carried out in more than one continent were denominated '*mixed*';

- Design used: cross-sectional, cross-sectional nested within a longitudinal study, and longitudinal;

- Age groups: all ages, children only, adolescents and adults, elderly only;

- Sample size: divided into ≤ 1000, 1001–5000, >5000 subjects;

- Sampling strategy: utilization of a random sampling strategy or not;

- Questionnaire administration mode: face-to-face interview, postal questionnaire, self-administration and telephone;

- Respondent: medicine user, medicine user and proxy, medicine user and parents, parents only, family breadwinner or any resident;

- Presentation of the survey questions that define the use of medicines in the article: yes or no;

- Presentation of data on the validity of the questionnaire used for evaluating medicine utilization: yes or no;

- Type of questions used to investigate medicine use: (a) open ended questions (in general, the first question is: "Have you used any medicine in the last xx days?" After a positive answer, a second group of questions includes identification and characterization of the medicines, following the specific objectives of the study); (b) checklists (questions about specific medicine names, pharmacological groups or diseases which are exclusively of interest to the study).

- Request for packaging presentation: yes or no;

- Request for prescription presentation: yes or no;

- Types of medicines investigated: medicines for chronic use, medicines for acute use or both;

- Indication of medicine taking: investigation of prescribed, over-the-counter or both types of medicines;

- Pharmacological group classification: classification system used for dividing medicines into pharmacological groups;

- Denominator used in the analyses: individuals, medicines or both;

- Recall period: as described in each article.

## Results

Additional file 1 summarizes the 61 studies included in our review, [4-64] whereas Additional files 2 and 3 present detailed characteristics of each article. Most studies were carried out in Europe and North America (64.0%), and used cross sectional designs. Sample size was greater than 1000 subjects in two-thirds of the studies, and most (95.1%) reported using random sampling strategies. More than 80% used interviewers to collect data. In the three studies including children,[32,49,62] the information on medicine use was provided by parents. In only 30% of the studies, the questions used to define medicine utilization were presented in the publication; in 93.4%, authors did not provide information on the validity of the questionnaire used to assess medicine use. Approximately 75% of studies used open ended questions, 59% required the presentation of the medicine packaging and only 15% required the presentation of medical prescriptions. In 11.5% of studies, only medicines for chronic illness were assessed, and 90% collected information about medicines regardless of being prescribed or not. The Anatomical Therapeutic Chemical (ATC) was the most frequent pharmacological group classification system used, although 42.5% of the studies did not indicate the classification system used. Most studies used individuals as the denominator in the analyses.

**Figure 1:**
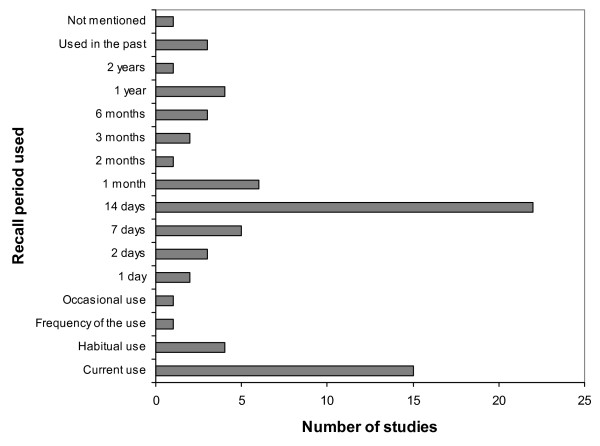
Distribution of the studies according to the recall period used.

The most frequently used recall period was 14 days, or two weeks, although the periods ranged from one day to two years (Figure 1). The recall period differed in studies investigating only medicines to treat chronic diseases (or for regular use)[5,28,34,50,51,56,59] compared to those evaluating medicines for acute use. The former usually asked about current use without a defined reference period. When both groups of medicines were investigated, some studies differentiated the recall period for each kind of medicine.[31,33,39,45,55]

## Discussion

Data on past utilization of medicines that are collected by interview may be inaccurate, since they are subject to imprecise recall (leading to omission of medicines used), incomplete information (for example, interviewees knowing the name of a medicine, but not the dose or duration of the treatment) or even wrong information (due to misunderstanding either the question itself or the medicine treatment in use) [33,64]. As a consequence, some methodological aspects are crucial for deriving valid information on medicine use through household surveys.

The following sections discuss several key methodological aspects of household surveys on medicine utilization, summarizing the strategies used in the reviewed studies, quantified in the results section, and presenting directions and recommendations for future studies in the field.

### 1. Recall Period

One of the most important methodological aspects in investigating medicine utilization through questionnaires is the definition of the recall period to be used. There is no consistency in the literature regarding the ideal recall period to investigate medicine utilization. However, it is known that the prevalence of medicine utilization is extremely dependent on the recall period. For example, two population-based studies carried out two years apart in Pelotas, Brazil, using similar methodologies, found prevalence of antimicrobial use in adults of 7.3% using a recall period of 30 days, and of 5.4% using a recall period of 15 days.[13] Another study carried out in Porto Alegre, Brazil [65] collected information about medicine utilization using two recall periods. The prevalence of medicine utilization 15 days prior to the interview was 54.5%, and in the day before the interview, it was 35.5%.

The main methodological issue related to the choice of the recall period is the difficulty in accurately remembering and reporting use within the period. Van den Brandt et al[66] showed that the accuracy of recall of used medicines decreases with increasing age and with increasing number of prescribed medicines taken, but the investigators did not find any recall differences between men and women. In addition to the general problem of inaccurate recall, a more serious problem can occur when two or more groups to be compared have different levels of recall accuracy. Sick people may report some variables (for example, medicine use) more accurately than healthy people.[55]

The main question to be answered when selecting recall period is: to what extent it is reasonable for the sample of respondents in this study to accurately recall details of medicines taken for the period in question? Difficulty in remembering is likely to be greater for longer period,[3] but the prevalence of medicine utilization is also likely to be greater due to accumulated medicine use during the period.[41] However, longer periods may lead to underreporting of some types of medicine, leading to underestimation of their use.[29,41]

Self-prescribed medicines are especially sensitive to difficulty at remembering.[3] A study carried out in Quenia[67] showed 60% underreporting of self-prescribed medicines when a two week recall period was used instead of one day. Medicines used regularly, such as cardiovascular medicines, may be more easily and accurately recorded using short recall periods.[27]

There is no ideal recall period for investigating medicine utilization, because selection of recall period depends on what is to be recalled and ability of the respondent population to remember. If the goal is to compare the data with most studies in the literature, two weeks (14–15 days) is the recommended period. There is a tendency in the literature that studies on acute illnesses use shorter recall periods in comparison to those on chronic illnesses.

### 2. Questionnaire administration

The procedure for administering the questionnaires (face-to-face, telephone, mail interview or self-application) is another important methodological aspect to be considered. A recent Brazilian study[4] about medicine utilization in the retired population compared two approaches for investigating medicine use: a postal self-completed questionnaire and a household questionnaire completed by interviewers. Response rates were twice as high in the face-to-face approach. In Europe, studies[11,68,69] reported postal response rates ranging from 71 to 92%; in the United States, published studies reported response rates as low as 60% [70] and 40% [71] for postal questionnaires.

In low and middle-income countries, telephone coverage may be quite low, and studies using this approach are likely to be affected by selection bias. High illiteracy rates, also frequent in less developed countries, make self-administration challenging. Finally, because response rates are quite low for postal questionnaires, studies using the face-to-face approach are recommended, particularly in low and middle-income countries.

### 3. Questions used to assess medicine utilization

Other relevant questions for household surveys on medicine utilization are the suitability of the terminology used,[29] the clear definition of the "medicine use" variable[72] and the length of the questionnaire. In many situations, technical terminology cannot be understood by the lay population, and particularly in settings with low levels of formal education, simple terminology should be used. The definition of what constitutes a medicine must be clear, considering that some medicines such as analgesics, natural products, medicines for topical use may not be reported by the respondents.[72]

In the study by Flores[27], it was noted that the level of information about the medicine concept was low. The interviewees frequently failed to report medicines to reduce weight, allergies, pain, diarrhea, kidney or bladder problems, digestive problems, colds or any kind of vitamins[27]. In terms of the length of the questionnaire, a study by Acurcio[4] suggested that the length of the postal questionnaire was one possible cause of low response rate.

In summary, researchers should use simple, but technically correct terminology. Efforts should be made to state clearly the definition of medicines to be used in the study, and special attention should be paid to prompting respondents for information about analgesics, natural products, medicines for topical use, and others that they may consider to be 'less important'.

### 4. Types of questions for investigating medicine utilization

Kimmel et al[73] studied the effect of reading a list of medicines and the presentation of photos as a prompt to recall the used medicines in a case-control study, which resulted in a 6% increase in the previously recalled medicines. Flores and Mengue,[27] studying overall medicine utilization in the elderly, found a prevalence of 86% using an open ended question, and 91% using a checklist.

Open ended questions are preferred for studies evaluating overall medicine utilization. Checklists should be used when authors are particularly interested in a given disease, pharmacological group or specific medicine names. The question(s) used for assessing medicine utilization should be presented in the articles, so that other researchers can replicate the method in future studies. Unfortunately, the majority of studies reviewed did not present the main question used to assess medicine utilization.

### 5. Implications of using information provided by relatives or caregivers

Because most studies on medicine utilization include elderly subjects, methodological attempts to improve the accuracy of the information on medicine use are a priority. Medicine use of the elderly differs considerably from other age groups.[74] The high number of medicines used, hearing and visual impairments, and lack of memory may reduce the quality of the information given. More serious morbidities may preclude direct interview. Some studies decided to exclude the subjects not able to answer the questionnaire.[13]

In order to minimize this problem, some researchers have opted to interview close relatives or caregivers for the total or partial collection of information about medicine utilization.[75] In some situations, such information may be more reliable and valid.[30] One should note that only 26.3% of the studies including only elderly subjects opted to use this strategy. However, other publications may simply have failed to report this kind of information about the data collection process.

Excluding elderly subjects without ability to answer a questionnaire on medicine utilization will underestimate the prevalence of medicine use, because these subjects are more likely to have medicine utilization. Therefore, the utilization of proxy information is recommended, particularly in studies with elderly subjects. Finally, authors should mention in the articles whether or not this strategy was used, so that other researchers would be able to replicate the methodology used, and correctly interpret the findings of the study.

### 6. Packaging and prescription presentation

One way to validate the data on medicine utilization and even to add recall about what was used in a specific period is to ask for the medicine packaging. This strategy is particularly useful in household studies, where, medicines tend to be stocked. As presented in Additional file 1, the majority of the studies reviewed asked for the packaging presentation. In some studies, the interviewees were asked to show all the used medicines at the moment or in the past. The interviewer had the task of selecting the eligible medicines for the study. In other cases, the interviewers asked only for the presentation of the medicines concerned with the study.

The request to see prescriptions may also be used to validate or qualify the data collected, because researchers may be able to correctly identify the name and characteristics of the medicines taken. In spite of its potential benefits, this strategy was used in only 15% of the studies reviewed.

### 7. Medicines for regular use and medicines used occasionally

Most household surveys on medicine utilization investigated medicines for treating both chronic and acute diseases. However, characterization of what constitutes regular use differs across studies. Some approaches are: use of medicine habitually;[55] use of medicines on a regular basis or continuously;[39,45] current use;[16,31,33] medicines used with fixed-time interval or for repetitive disease occurrence;[51] medicines to chronic diseases;[50] and medicines used regularly, without a date to stop.[13]

Considering that the terminology used may affect the interviewee's answer, it is important to clarify exactly what is intended. For example, oral contraceptives are medicines used regularly, but they are not used to treat chronic diseases as the majority of the other medicines included in this group.

Another issue to consider is the duration of time using a medicine in order to it to be considered chronically or habitually used. Some prophylactic treatments with antibiotics are long, but temporary. Should they be considered for chronic or acute use? In this sense, the utilization of terms like "habitual" or "chronic" seem to be more appropriate than the definition based on the number of days in treatment. The term "without date to stop" may be a useful alternative to differentiate the types of medicines.

### 8. Pharmacological group classification

When the study requires a classification into pharmacological groups, the quality of the information is a concern, and presentation of the medicine packaging or prescription is a useful practice.[76] Almost half of the reviewed studies did not indicate the system used to classify the medicines by pharmacological groups.

Many medicines have two or more indications; in order to classify medicines into pharmacological groups, it is essential to know which health problem the medicine was used for. In the case of acetylsalicylic acid, for example, the ATC code N02BA01 would identify use as an analgesic, while B01AC06 would identify use as a platelet aggregation inhibitor.

There is no ideal system for classifying medicines into pharmacological groups. However, it is extremely important that authors mention the system chosen, in order to allow comparisons across studies.

### 9. Denominators used in the analyses

Medicine utilization may be analyzed using either the total number of subjects in the sample or the total number of reported medicines as the denominator, which can lead to very different conclusions about the prevalence of use of different types of medicines. Some studies do not inform about which kind of denominator was used in the analyses, making it difficult to interpret the data or compare across studies.

In studies of individual determinants of medicine utilization, the denominator should be the number of subjects in the sample. In studies on the characteristics of the medicines used, both approaches have been used. In the first approach, each line in the database will correspond to a subject, while in the second one, each line in the database corresponds to a medicine used. This aspect was recently discussed in a review paper.[72]

In studies of specific pharmacological groups, one may be interested in reporting the prevalence of antimicrobial use in the studied population (denominator = number of individuals) or the proportion of antimicrobials in the total number of medicines used in the sample (denominator = number of medicines).

In a study about utilization of generic medicines,[77] both approaches resulted in very similar values; 3.9% (over the total of medicines) and 3.6% (over the total of subjects in the sample). However, this is not always the case. Bertoldi et al. [13] found that 16.5% of the medicines used were non-opioid analgesics. However, 32.2% of the respondents used at least one non-opioid analgesic in the period investigated. These findings show the importance of the denominator in the interpretation of the studies, and highlight the need to state clearly which denominator is used in each analysis.

## Conclusion

The present review focused on the methods used in 61 household surveys of medicine utilization published between January 1995 and June 2008. These data will be useful for updating researchers on current standards of practice, and may help overcome challenges in the planning and analyses of such studies. Moreover, recommendations in this publication may help to improve the quality of the data about medicine use obtained in household surveys.

Given that studies are conducted for different purposes, a certain degree of heterogeneity in methods is expected. However, standardization of certain practices would be beneficial. For example, international and regional comparisons of medicine utilization prevalence would benefit from the utilization of consistent methodologies. Actually, the World Health Organization has tried to overcome the problem, by proposing standard instruments. In the 2002 World Health Survey, a standardized questionnaire including some questions on medicine access, affordability and use, was administered in 53 countries[78].

Regardless of the issue of standardization, some publications lack important details on the methodologies used, for example, the system used to classify medicines into pharmacological groups or the question used to investigate the main outcome variable. Authors should provide such details in the articles, so that other researchers may adequately replicate studies in different settings. Finally, because data on the reliability and validity of medicine use questionnaires are rare, it is uncertain if the instruments currently in use are accurately assessing the construct of medicine utilization.

## Competing interests

The authors declare that they have no competing interests.

## Authors' contributions

ADB led the literature review process, the writing and analyses of the manuscript. AJDB, AW and DR-D approved the search strategy, revised the text and contributed in the planning of the review. PCH conducted the literature review with ADB, assessed each article in terms of the inclusion criteria and helped writing the manuscript and analyzing the data.

## Pre-publication history

The pre-publication history for this paper can be accessed here:



## Supplementary Material

Additional file 1 **Table 1.** Quantification of methodological characteristics of the 61 household surveys on medicine utilization included in the review. Click here for file

Additional file 2 **Table 2.** Description of the studies included in the review according to methodological characteristics – part I. Click here for file

Additional file 3 **Table 3.** Description of the studies included in the review according to methodological characteristics – part II. Click here for file
