# Supplementary material for: A descriptive review of the methodologies used in household surveys on medicine utilization
Source: BMC Health Serv Res. 2008 Oct 31;8:222. doi: 10.1186/1472-6963-8-222 (PMC2584639; doi:10.1186/1472-6963-8-222)
Supplement: Additional file 1 — Table 1. Quantification of methodological characteristics of the 61 household surveys on medicine utilization included in the review. [file 1472-6963-8-222-S1.doc]

TABLE 1. Quantification of methodological characteristics of the 61 household surveys on medicine utilization included in the review.

| **Characteristics** | **N** | **%** |
| --- | --- | --- |
| Continent |  |  |
| Europe | 25 | 41.0 |
| North America | 14 | 23.0 |
| South America | 12 | 19.7 |
| Asia | 5 | 8.2 |
| Africa | 3 | 4.9 |
| Oceania | 1 | 1.6 |
| Mixed^1^ | 1 | 1.6 |
| Design |  |  |
| Cross-sectional | 47 | 77.0 |
| Cross-sectional nested in longitudinal | 9 | 14.8 |
| Longitudinal | 5 | 8.2 |
| Age group |  |  |
| All | 8 | 13.1 |
| Children | 3 | 4.9 |
| Adolescents and adults | 19 | 31.2 |
| Only elderly | 31 | 50.8 |
| Sample size |  |  |
| ≤ 1000 | 20 | 32.8 |
| From 1001 to 5000 | 27 | 44.2 |
| > 5000 | 14 | 23.0 |
| Utilization of a random sampling strategy |  |  |
| No | 3 | 4.9 |
| Yes | 58 | 95.1 |
| Questionnaire administration |  |  |
| Interviewer | 50 | 82.0 |
| Mail | 5 | 8.2 |
| Self-administered | 3 | 4.9 |
| Interviewer + mail | 2 | 3.3 |
| Telephone | 1 | 1.6 |
| Interview respondent |  |  |
| Medicine users | 38 | 62.4 |
| Medicine users and by proxy^2^ | 13 | 21.3 |
| Medicine user and parents | 5 | 8.2 |
| Parents | 3 | 4.9 |
| Family breadwinner | 1 | 1.6 |
| Any resident | 1 | 1.6 |
| Presentation of the question used to investigate medicine use in the publication |  |  |
| No | 43 | 70.5 |
| Yes | 18 | 29.5 |
|  |  |  |

| Presentation of data on the validity of the questionnaire used to assess medicine use |  |  |
| --- | --- | --- |
| No | 57 | 93.4 |
| Yes | 4 | 6.6 |
| Type of questions used to investigate medicine use |  |  |
| Open ended question | 46 | 75.5 |
| Other kinds of questions^3^ | 8 | 13.1 |
| Checklist | 6 | 9.8 |
| Not mentioned | 1 | 1.6 |
| Request for the packaging presentation |  |  |
| No | 20 | 32.8 |
| Yes | 36 | 59.0 |
| NA^4^ | 5 | 8.2 |
| Request for the prescription presentation |  |  |
| No | 47 | 77.0 |
| Yes | 9 | 14.8 |
| NA^4^ | 5 | 8.2 |
| Types of medicine studied |  |  |
| Prescribed and over-the-counter medicines | 55 | 90.2 |
| Only prescribed medicines | 3 | 4.9 |
| Only over-the-counter | 3 | 4.9 |
| Types of medicine use |  |  |
| Chronic and acute use | 54 | 88.5 |
| Only chronic use | 7 | 11.5 |
| Pharmacological group classification |  |  |
| ATC^5^ | 14 | 23.0 |
| AHF^6^ | 4 | 6.6 |
| Other^7^ | 14 | 23.0 |
| Not mentioned | 26 | 42.5 |
| NA^4^ | 3 | 4.9 |
| Denominator used in the analyses |  |  |
| Individuals | 39 | 64.0 |
| Individuals and medicines | 21 | 34.4 |
| Not mentioned | 1 | 1.6 |

^1^ Mexico, Philippines, Uganda and the United States

^2^ Caregivers or close relatives

^3^ Part open ended and part checklist (5 cases); open ended, but induced (1 case); specified list of medical conditions (2 cases)

^4^ Not applicable

^5^ Anatomical Therapeutic Chemical Classification System

^6^ American Hospital Formulary System

^7^ Other classification, which was presented only once
