# Supplementary material for: A descriptive review of the methodologies used in household surveys on medicine utilization
Source: BMC Health Serv Res. 2008 Oct 31;8:222. doi: 10.1186/1472-6963-8-222 (PMC2584639; doi:10.1186/1472-6963-8-222)
Supplement: Additional file 2 — Table 2. Description of the studies included in the review according to methodological characteristics – part I. [file 1472-6963-8-222-S2.doc]

TABLE 2. Description of the studies included in the review according to methodological characteristics – part I.

| **1st author**  **(year of publication)** | **Country** | **Design** | **Age**  **(yr)** | **N**^1^ | **Questionnaire administration** | **Interview respondent** | **Presentation of the question used to assess medicine use** |
| --- | --- | --- | --- | --- | --- | --- | --- |
| Acurcio (2006) | Brazil | Cross-sectional | >59 | 1025 | Interviewer/mail | Subject and proxy | No |
| Agostini (2004) | US | Longitudinal^2^ | >71 | 885 | Interviewer | Subject | No |
| Al-Windi (2000) | Sweden | Cross-sectional | >15 | 1312 | Mail | Subject | Yes |
| Amare (1997) | Ethiopia | Cross-sectional | All | 903 | Interviewer | Subject and parents | Yes |
| Arrais (2005) | Brazil | Cross-sectional | All | 1366 | Interviewer | Subject and parents | No |
| Awad (2006) | Sudan | Cross-sectional | >17 | 1200 | Self-administered | Subject | No |
| Barat (2000) | Denmark | Cross-sectional | 75 | 492 | Interviewer | Subject | No |
| Bardel (2000) | Sweden | Cross-sectional | 35-65 | 2991 | Mail | Subject | No |
| Barros e Sá (2007) | Brazil | Cross-sectional | >59 | 355 | Interviewer | Subject | No |
| Bertoldi (2004) | Brazil | Cross-sectional | >19 | 3182 | Interviewer | Subject | Yes |
| Blalock (2005) | US | Cross-sectional | >64 | 800 | Interviewer | Subject | No |
| Brekke (2006) | Norway | Cross-sectional | 70-74 | 3341 | Mail | Subject | Yes |
| Brzozowska (2002) | 9 countries^3^ | Longitudinal | 75-86 | 621 | Interviewer | Subject | No |
| Carrasco-Garrido (2008) | Spain | Cross-sectional | >15 | 19514 | Interviewer | Subject | Yes |
| Carvalho (2005) | Brazil | Cross-sectional | >17 | 5000 | Interviewer | Subject | No |
| Chen (2001) | 2 countries^4^ | Cross-sectional | >64 | 12489 | Computer^5^ | Subject | Yes |
| Coelho Filho (2004) | Brazil | Cross-sectional | >59 | 697 | Interviewer | Subject | No |
| Cohen (1998) | Australia | Cross-sectional | >59 | 1611 | Mail | Subject | No |
| Del Rio (1997) | Spain | Cross-sectional | All | 26334 | Interviewer | Subject and parents | Yes |
| Eggen (1997) | Norway | Cross-sectional | 0-80 | 10576 | Interviewer | Subject and parents | No |
| Espino (1998) | US | Cross-sectional | >64 | 2899 | Interviewer | Subject | No |
| Figueiras (2000) | Spain | Cross-sectional | >15 | 20311 | Interviewer | Subject | No |
| Fillenbaum (1996) | US | Longitudinal | >64 | 3224 | Interviewer | Subject and proxy | No |
| Flores (2005) | Brazil | Cross-sectional | >59 | 215 | Interviewer | Subject | No |
| Fuchs (2003) | Israel | Longitudinal^2^ | >74 | 1369 | Interviewer | Subject and proxy | No |
| Furu (1997) | Norway | Cross-sectional | 20-62 | 15986 | Self-administered | Subject | Yes |
| Gama (1998) | Spain | Cross-sectional | >64 | 362 | Interviewer | Subject and proxy | Yes |
| Hach (2004) | Germany | Longitudinal^2^ | 18-24 | 2064 | Interviewer | Subject | No |
| Headley (2007) | UK | Longitudinal^2^ | 0-7,5 | 14062 | Mail | Parents | Yes |
| Hershman (1995) | US | Longitudinal | 75-85 | 488 | Interviewer | Subject | No |
| Hidalgo (1997) | Spain | Cross-sectional | >64 | 1015 | Interviewer | Subject | No |
| Hogan (1995) | Canada | Cross-sectional | >64 | 10263 | Interviewer | Subject and proxy | No |
| Izazola-Conde (1998) | Mexico | Cross-sectional | All | 209938 | Interviewer | Subject | Yes |
| Johnson (1996) | US | Cross-sectional | >18 | 5059 | Interviewer/mail | Subject | Yes |
| Kaufman (2002) | US | Cross-sectional | >17 | 2590 | Telephone | Subject and proxy | No |
| Klarin (2003) | Sweden | Longitudinal^2^ | >74 | 1671 | Interviewer | Subject | No |
| Lassila (1996) | US | Cross-sectional | >64 | 1360 | Interviewer | Subject and proxy | No |
| Loyola Filho (2002) | Brazil | Cross-sectional | >17 | 775 | Interviewer | Subject and proxy | Yes |
| Loyola Filho (2006) | Brazil | Cross-sectional | >59 | 1598 | Interviewer | Other resident | No |
| Miralles (1998) | Brazil | Cross-sectional | >59 | 436 | Interviewer | Subject | No |
| Moxey (2003) | US | Longitudinal | >64 | 7798 | Computer^5^ | Subject and proxy | No |
| Nielsen (2003) | Denmark | Cross-sectional | >15 | 16690 | Interviewer | Subject | Yes |
| Obermeyer (2002) | Lebanon | Cross-sectional | 25-64 | 14142 | Interviewer | Subject and proxy | Yes |
| Obermeyer (2004) | 4 countries^6^ | Cross-sectional | >14 | 539 | Interviewer | Subject | No |
| Obermeyer (2007) | US | Cross-sectional | 15-81 | 80 | Interviewer | Subject | No |
| Quiroga (1996) | Spain | Cross-sectional | >60 | 825 | Interviewer | Subject and proxy | No |
| Rajmil (2000) | Spain | Cross-sectional | <15 | 2433 | Interviewer | Parents | No |
| Recalde (1998) | Spain | Cross-sectional | >65 | 1284 | Interviewer | Subject | Yes |
| Rosholm (1997) | Denmark | Longitudinal | >75 | 2171 | Interviewer | Subject | No |
| Sans (2002) | Spain | Cross-sectional | 25-64 | 3421 | Interviewer | Subject | No |
| Shankar (2003) | Nepal | Cross-sectional | All | 164 | Interviewer | Household head^7^ | No |
| Shankar (2002) | Nepal | Cross-sectional | >10 | 142 | Interviewer | Subject | No |
| Simoni (2000) | Italy | Longitudinal^2^ | 8-73 | 1946 | Interviewer | Subject | No |
| Steyn (2005) | South Africa | Cross-sectional | >15 | 13826 | Interviewer | Subject | No |
| Stoehr (1997) | US | Cross-sectional | >64 | 1059 | Interviewer | Subject and proxy | Yes |
| Thomas (1999) | Wales | Longitudinal^2^ | 56-75 | 1906 | Self-administered | Subject | No |
| Vilarino (1998) | Brazil | Cross-sectional | All | 413 | Interviewer | Subject and parents | No |
| Wallsten (1995) | US | Cross-sectional | 70-79 | 242 | Interviewer | Subject | Yes |
| Weiderpass (1998) | Brazil | Longitudinal^2^ | 1-3 ^8^ | 644 | Interviewer | Parents | No |
| Wills (1997) | Sweden | Longitudinal^2^ | >75 | 1700 | Interviewer | Subject and proxy | No |
| Woo (1995) | China | Cross-sectional | >70 | 2032 | Interviewer | Subject | No |

^1^ N=Sample size ^2^ Cross-sectional studies nested within longitudinal studies were classified as longitudinal

^3^ Belgium, Denmark, France, Italy, Netherlands, Portugal, Spain, Switzerland, Poland. ^4^ England and Wales

^5^ Personal interview assisted by computer ^6^ Mexico, Philippines, Uganda, US

^7^ Oldest female living in the household in most cases ^8^ Age in months
