# Supplementary material for: A descriptive review of the methodologies used in household surveys on medicine utilization
Source: BMC Health Serv Res. 2008 Oct 31;8:222. doi: 10.1186/1472-6963-8-222 (PMC2584639; doi:10.1186/1472-6963-8-222)
Supplement: Additional file 3 — Table 3. Description of the studies included in the review according to methodological characteristics – part II. [file 1472-6963-8-222-S3.doc]

TABLE 3. Description of the studies included in the review according to methodological characteristics – part II.

| **1st author**  **(year of publication)** | **Type of question** | **Packaging request** | **Prescription request** | **Types of medicines**^1^ | **Type of use**^2^ | **Denominator used**^3^ | **Pharmacological group classification** |
| --- | --- | --- | --- | --- | --- | --- | --- |
| Acurcio (2006) | Open ended | Yes | Yes | Both | Both | NA^4^ | Other^5^ |
| Agostini (2004) | Open ended | Yes | No | Both | Chronic | Individuals | AHF^6^ |
| Al-Windi (2000) | Open ended | NA^4^ | NA^4^ | Both | Both | Individuals | NA^4^ |
| Amare (1997) | Open ended | Yes | No | Both | Both | Individuals | Not mentioned |
| Arrais (2005) | Open ended | Yes | Yes | Both | Both | Individuals | NA |
| Awad (2006) | Other^8^ | No | No | Over-the-counter | Both | Individuals | Not mentioned |
| Barat (2000) | Open ended | Yes | No | Both | Both | Both | ATC^7^ |
| Bardel (2000) | Open ended | NA^4^ | NA^4^ | Prescribed | Both | Individuals | ATC^7^ |
| Barros e Sá (2007) | Open ended | No | No | Over-the-counter | Both | Both | Other^5^ |
| Bertoldi (2004) | Open ended | Yes | Yes | Both | Both | Both | Other^5^ |
| Blalock (2005) | Open ended | Yes | No | Both | Both | Individuals | Other^5^ |
| Brekke (2006) | Open ended | No | No | Both | Both | Both | ATC^7^ |
| Brzozowska (2002) | Open ended | No | No | Both | Both | Individuals | Not mentioned |
| Carrasco-Garrido (2008) | Other^8^ | No | No | Both | Both | Individuals | Not mentioned |
| Carvalho (2005) | Open ended | Yes | Yes | Both | Both | Individuals | Other^5^ |
| Chen (2001) | Open ended | Yes | No | Both | Both | Individuals | Other^5^ |
| Coelho Filho (2004) | Open ended | Yes | No | Both | Both | Individuals | ATC^7^ |
| Cohen (1998) | Not mentioned | NA^4^ | NA^4^ | Both | Both | Individuals | Not mentioned |
| Del Rio (1997) | Open ended | No | No | Both | Both | Both | Not mentioned |
| Eggen (1997) | Open ended | No | No | Both | Both | Individuals | NA^4^ |
| Espino (1998) | Open ended | Yes | No | Both | Both | Individuals | Other^5^ |
| Figueiras (2000) | Open ended | No | No | Both | Both | Individuals | Not mentioned |
| Fillenbaum (1996) | Open ended | Yes | No | Both | Both | Individuals | Other^5^ |
| Flores (2005) | Other^8^ | Yes | No | Both | Both | Both | ATC^7^ |
| Fuchs (2003) | Checklist | Yes | No | Both | Chronic | Both | Other^5^ |
| Furu (1997) | Other^8^ | NA^4^ | NA^4^ | Both | Both | Individuals | Not mentioned |
| Gama (1998) | Open ended | Yes | No | Both | Both | Individuals | Not mentioned |
| Hach (2004) | Open ended | Yes | No | Both | Both | Both | ATC^7^ |
| Headley (2007) | Other^8^ | No | No | Both | Both | Both | ATC^7^ and other^5^ |
| Hershman (1995) | Open ended | Yes | Yes | Both | Both | Individuals | Not mentioned |
| Hidalgo (1997) | Open ended | Yes | No | Both | Chronic | Individuals | Other^5^ |
| Hogan (1995) | Open ended | No | No | Both | Both | Individuals | AHF |
| Izazola-Conde (1998) | Open ended | No | No | Both | Both | Both | Not mentioned |
| Johnson (1996) | Checklist | NA^4^ | NA^4^ | Over-the-counter | Both | Individuals | Not mentioned |
| Kaufman (2002) | Open ended | Yes | No | Both | Both | Both | Other^5^ |
| Klarin (2003) | Open ended | Yes | Yes | Both | Both | Individuals | ATC^7^ |
| Lassila (1996) | Open ended | Yes | No | Both | Both | Individuals | AHF^6^ |
| Loyola Filho (2002) | Open ended | No | No | Both | Both | Both | Not mentioned |
| Loyola Filho (2006) | Open ended | Yes | Yes | Both | Both | Both | ATC^7^ |
| Miralles (1998) | Open ended | Yes | No | Both | Both | Individuals | Not mentioned |
| Moxey (2003) | Open ended | Yes | Yes | Prescribed | Both | Individuals | Other^5^ |
| Nielsen (2003) | Other^8^ | No | No | Both | Both | Individuals | Not mentioned |
| Obermeyer (2002) | Checklist | No | No | Both | Both | Individuals | Not mentioned |
| Obermeyer (2004) | Other^8^ | No | No | Both | Both | Individuals | Not mentioned |
| Obermeyer (2007) | Checklist | Yes | No | Both | Both | Both | AHF^6^ |
| Quiroga (1996) | Open ended | Yes | No | Both | Chronic | Both | Not mentioned |
| Rajmil (2000) | Open ended | No | No | Both | Both | Individuals | Not mentioned |
| Recalde (1998) | Other^8^ | Yes | No | Both | Chronic | Individuals | ATC^7^ |
| Rosholm (1997) | Open ended | No | No | Both | Chronic | Individuals | ATC^7^ |
| Sans (2002) | Open ended | Yes | Yes | Both | Both | Both | ATC^7^ |
| Shankar (2003) | Open ended | Yes | No | Both | Both | Individuals | Not mentioned |
| Shankar (2002) | Open ended | Yes | No | Both | Both | Both | Not mentioned |
| Simoni (2000) | Open ended | No | No | Both | Both | Both | Other^5^ |
| Steyn (2005) | Checklist | Yes | No | Prescribed | Chronic | Individuals | ATC^7^ |
| Stoehr (1997) | Open ended | Yes | No | Both | Both | Individuals | Not mentioned |
| Thomas (1999) | Open ended | No | No | Both | Both | Individuals | Other^5^ |
| Vilarino (1998) | Open ended | No | No | Both | Both | Both | Not mentioned |
| Wallsten (1995) | Open ended | Yes | No | Both | Both | Both | Not mentioned |
| Weiderpass (1998) | Open ended | Yes | No | Both | Both | Both | Not mentioned |
| Wills (1997) | Open ended | Yes | No | Both | Both | Individuals | ATC^7^ |
| Woo (1995) | Checklist | Yes | No | Both | Both | Individuals | Not mentioned |

^1^ Types of medicines investigated in the survey: over-the-counter, prescribed or both

^2^ Type of medicine use investigated in the survey: to treat chronic diseases, to treat acute diseases or both

^3^ Denominators used in the analyses: individuals, medicines or both.

^4^ NA: Not applicable

^5^ Other classification that was mentioned only once

^6^ AHF: American Hospital Formulary System

^7^ Anatomical Therapeutic Chemical Classification System

^8^  Mix of open ended questions and checklists or a specific list of medical conditions.
